# Evaluation of Pro/Antioxidant Imbalance in Blood of Women with Polycystic Ovary Syndrome Based on Determination of Oxidized Low-Density Lipoproteins and Ferric Reducing Ability of Plasma Values

**DOI:** 10.3390/biomedicines10071564

**Published:** 2022-06-30

**Authors:** Justyna Niepsuj, Grzegorz Franik, Paweł Madej, Agnieszka Piwowar, Anna Bizoń

**Affiliations:** 1 Students Scientific Society at the Department of Toxicology, Faculty of Pharmacy, Wroclaw Medical University, 50-556 Wroclaw, Poland; justyna.niepsuj@student.umw.edu.pl; 2 Department of Endocrinological Gynecology, Medical University of Silesia, 40-752 Katowice, Poland; pfranik@sum.edu.pl (G.F.); pmadej@sum.edu.pl (P.M.); 3 Department of Toxicology, Faculty of Pharmacy, Wroclaw Medical University, 50-556 Wroclaw, Poland; agnieszka.piwowar@umw.edu.pl

**Keywords:** polycystic ovary syndrome, pro/antioxidant balance, oxidized low-density lipoproteins, antioxidant status, ferric reducing ability of plasma

## Abstract

We investigated selected pro/antioxidant parameters in a group of women with polycystic ovary syndrome (PCOS) divided according to age, body mass index (BMI), waist-to-hip ratio (WHR), homeostatic model assessment for insulin resistance (HOMA-IR) and quantitative insulin sensitivity check index (Quicki). We chose oxidized low-density lipoproteins (oxLDL-C) as a marker of oxidative stress and the ferric reducing ability of plasma (FRAP) as a marker of antioxidant status. In women with PCOS, the values of BMI, WHR, age and concentration of glucose significantly affected oxLDL-C concentration and FRAP values. FRAP correlated with oxLDL-C level in the whole group and in women who were insulin sensitive (HOMA-IR < 2.0). There was a negative relationship between the concentration of Anti-Müllerian hormone and both oxLDL-C and FRAP. Furthermore, the value of FRAP was inversely correlated with luteinizing hormone (LH), follicle-stimulating hormone (FSH) and androstenedione, whereas it was positively correlated with the LH/FSH ratio. The concentration of oxLDL and the value of FRAP are significantly associated with selected metabolic and hormonal parameters in the course of PCOS.

## 1. Introduction

Polycystic ovary syndrome (PCOS) is a common reproductive and metabolic disorder affecting women of reproductive age [1]. According to the Rotterdam criteria, it is diagnosed when at least two out of three characteristics are met: clinical or biochemical hyperandrogenism, chronic ovulatory dysfunction and polycystic ovarian morphology [2]. The prevalence of this disorder ranges from 6% to 21% [3]. PCOS is often associated with obesity, hyperinsulinemia, insulin resistance (IR), dyslipidemia and higher cardiovascular risk [4]. The pathogenesis of this syndrome is still unknown; however, it seems that development of the disease results from the interaction between genetic and environmental factors, which can induce and intensify these disorders [5]. Additionally, the magnitude of oxidative stress (OS) could be treated as one of the most important agents influencing both the development and the course of PCOS [6,7]. Previous studies have shown that oxidative stress is significantly increased in blood and follicular fluid of women with PCOS compared with healthy controls [8,9]. It is possible that oxidative stress could affect steroidogenesis in the ovaries, which contributes to androgen excess, incorrect follicular development and infertility [10]. Moreover, the reactivity of oxygen species (ROS) was pointed as a cause of genetic changes such as point mutations, DNA strand breaks, aberrant DNA cross-linking, DNA–protein cross-linking and DNA methylation, as well as a possible cause of PCOS development [11]. Furthermore, oxidative stress is linked to all the main features of PCOS, such as obesity, IR, hyperandrogenism, follicular apoptosis and infertility [12]. ROS also induces oxidative damage to lipids and proteins [13]. Since cholesterol is a precursor of steroid hormones, a disturbed lipid profile is associated with abnormalities in the concentration of sex hormones, and thus contributes to disorders observed in the course of PCOS [14]. Therefore, in this study, as a marker of oxidative stress, we chose oxidized low-density lipoprotein (oxLDL-C), which occurs as a result of the reaction of oxygen free radicals with polyunsaturated fatty acids and lipid peroxidation in low-density lipoprotein (LDL-C) components [15]. OxLDL increases the oxLDL-dependent lectin-like oxidized low-density lipoprotein receptor-1 in endothelial cells, which causes an extension of ROS production. This process leads to apoptosis of endothelial cells and autophagy of granulosa cells [16]. OxLDL-C is also a known maker of atherosclerosis, and it was shown that both obese and non-obese women with PCOS have a higher concentration of oxLDL [17]. To counteract the harmful effects of oxidative stress, there are physiological protective mechanisms that provide antioxidant defense. They include enzymes and non-enzymatic molecules which can dispose and suppress ROS generation [18]. The combined ability of these compounds to remove ROS represents antioxidant capacity, which is crucial for maintaining balance between pro- and antioxidants. Antioxidant capacity of biological samples can be measured as concentrations of various antioxidants individually or as total antioxidant capacity (TAC) of serum/plasma, which estimates the antioxidant components of a sample in a global way. The second approach is much more convenient, because it requires less effort and is not that costly or time consuming [19,20]. There are several methods that can be used to evaluate TAC, for example, the Trolox equivalent antioxidant capacity (TEAC) assay, total radical trapping parameter (TRAP) and ferric reducing ability of plasma (FRAP) assay; for the purposes of this study, we chose to use the latter [20]. We assessed the differences in FRAP value and oxLDL-C concentration in subgroups divided according to important factors that seem to influence the course of PCOS. As insulin resistance is associated with PCOS, we divided our group according to the homeostatic model assessment for insulin resistance (HOMA-IR), considering 2.0 as a cut-off value [21]. Moreover, we also used the quantitative insulin sensitivity check index (Quicki) to separate an insulin sensitive subgroup from the patients who were not insulin sensitive. Our previous study showed a connection between waist-to-hip ratio (WHR) and hormone status [22]; therefore, we also divided the group according to the WHR. Other division criteria were age and body mass index (BMI). The aim of this study was to determine the concentration of oxLDL-C and the value of FRAP in described subgroups of PCOS patients and to assess PCOS’ association with hormone and lipid profile parameters.

## 2. Materials and Methods

The study was conducted on a group of 38 women who were hospitalized in the Gynecological Endocrinology Clinic of the Silesian Medical University in Katowice, Poland in 2021. PCOS was diagnosed using the Rotterdam criteria. Moreover, PCOS phenotypes were recognized: phenotype 1, with clinical and/or biochemical hyperandrogenism, oligomenorrhea and polycystic ovaries (*n* = 23); phenotype 2, with clinical and/or biochemical hyperandrogenism and oligomenorrhea (*n* = 3); phenotype 3, with clinical and/or biochemical hyperandrogenism and polycystic ovaries (*n* = 8); and phenotype 4, with oligomenorrhea and polycystic ovaries (*n* = 4). Patients exposed to tobacco smoke and alcohol abuse were excluded from the study. Hypertension, Cushing’s syndrome and adrenal tumors were also exclusion criteria. The assessment of 17α-hydroxyprogesterone (17-OHP) was performed to exclude nonclassical congenital adrenal hyperplasia (NCAH). To eliminate hyperprolactinemia, the concentration of prolactin was measured. The concentration of TSH was assayed to exclude hypothyroidism.

At the beginning, our study group consisted of 79 women. After applying the exclusion criteria, 38 patients were left. Blood samples were collected in the morning after an overnight fast (>12 h) till the 8th day of the menstrual cycle (follicular phase), according to standard procedures. Samples were immediately centrifuged. The remaining serum was frozen in −80 °C and oxidative stress parameters were assayed immediately after thawing. Lipid, hormonal and glucose metabolism parameters were assayed in fresh blood during hospitalization. Ethical approval was obtained from the Bioethical Committee of the Wroclaw Medical University (KBN No. 254/2021).

### 2.1. Oxidative Stress Parameters

OxLDL concentration was assayed using a commercially available ELISA kit (Mercodia AB, Cat. No.: 10-1143-01, Uppsala, Sweden) with the lower limit of sensitivity at 0.6 mU/L. The concentration of oxLDL in serum (a final dilution 1:6561) was assayed using a competitive ELISA method based on the direct sandwich procedure in which two monoclonal antibodies were directed against separate antigenic determinants on the oxidized apolipoprotein B-100. During 2 h incubation at 25 °C, oxLDL in serum reacted with antioxidized LDL antibodies coated on the wells. After washing (6 times with 350 µL of wash buffer), a peroxidase conjugated anti-human apolipoprotein B antibody, which recognizes oxLDL bound to the wells, was added. After second incubation (1 h incubation at 25 °C) and washing (6 times with 350 µL of wash buffer), the bound conjugate was detected by reaction with 3,3′,5,5′-tetramethylbenzidine (TMB). Then, the reaction was stopped and the absorbance was measured at λ = 450 nm. The absorbance was assayed at λ = 450 nm using a Synergy HTX multi-mode reader (S1LFA, BioTek, Santa-Clara, CA, USA). The inter-assay variation was 5.6% for the oxLDL ELISA and all samples were within detection range.

The FRAP assay was conducted using the colorimetric method with ferric tripyridyltriazine (FeIII-TPTZ) [20]. The method was based on the reduction of the FeIII-TPTZ complex to the ferrous form (FeII), which has an intense blue color. The FRAP reagent was prepared by mixing 300 mM acetate buffer (pH 3.6), 10 mM 2,4,6-tripyridyl-s-triazine (TPTZ) (Cat. No.: Acros 168070050, Thermo Scientific, Austria) in 40 mM HCl and 20 mM aqueous solution of FeCl_3_ × 6H_2_O (Cat. No.: 119041804, Chempur, Poland) in a proportion of 10:1:1. Samples were diluted in water (1:9) and 100 µL of sample was added to 500 µL of freshly prepared FRAP reagent, incubated for 5 min at 37 °C and then centrifuged at 2000× *g* for 10 min. The absorbance of the supernatants was measured at λ = 593 nm against a reagent blank using a visible spectrophotometer (SP-830 Plus, Metertech, Nankang, Taipei, Taiwan).

### 2.2. Glucose and Insulin Concentration and Lipid Profile Parameters

Plasma glucose concentration was assessed using the colorimetric method (Roche Diagnostics, Indianapolis, IN, USA). Serum insulin concentration was measured with an ELISA (DRG Instruments GmbH, Marburg, Germany). All women underwent an oral glucose tolerance test (OGTT) after ingestion of a solution of 75 g of glucose. The levels of total cholesterol (CHO), low-density lipoprotein (LDL-C), high-density lipoprotein (HDL-C) and triglycerides (TG) were determined using commercially available test kits (Roche Diagnostics, Indianapolis, IN, USA). All the above parameters were measured during routine diagnostic determination.

### 2.3. Hormone Assay

The concentration of follicle-stimulating hormone (FSH), luteinizing hormone (LH), total testosterone (total T), free testosterone (free T), androstenedione (AD), dehydroepiandrosterone sulfate (DHEA-S) and prolactin were determined by an ELISA (DRG Instruments GmbH, Marburg, Germany) with the lower limits of sensitivity at 0.86 IU/L, 1.27 IU/L, 0.083 µg/L, 0.002 ng/L, 0.019 µg/L, 0.044 mg/L and 0.35 µg/L, respectively. The respective intra- and inter-assay coefficients of variations were: 5.5% and 6.1% for FSH, 5.6% and 6.2% for LH, 3.6% and 7.1% for total T, 6.4% and 8.0% for free T, 6.5% and 10.2% for AD, 4.8% and 7.5% for DHEA-S, and 4.5% and 5.9% for prolactin. The 17-OHP was assayed by RIA (Diagnostic Products Corporation, Los Angeles, CA, USA) with lower detectable concentrations of 0.2 nmol/L. The respective inter- and intraassay coefficients of variation were 5.6% and 8.0% for 17-OHP. Thyrotropin (TSH) was determined by two Roche Cobas Elecsys 600 tests.

Anti-Müllerian hormone (AMH) was assayed using an ELISA (Immunotech a.s., Prague, Czech Republic) with lower detectable concentrations of 0.05 ng/mL, and inter- and intra- assay coefficient of variations were 4.6% and 4.6%, respectively.

### 2.4. Calculated Parameters

For the estimation of insulin resistance, the homeostasis model for insulin resistance index (HOMA-IR) was calculated by the subsequent formula: [HOMA-IR = fasting glucose (mmol/L) × fasting insulin (µU/mL)/22.5)] [23].

Quicki was calculated according to the formula described earlier [QUICKI = 1/[log fasting insulin (µU/mL) + log fasting glycemia (mg/dL = mmol/L × 18.18)] [24].

Castelli risk index I (TC/HDL-C ratio) and Castelli risk index II (LDL-C/HDL-C ratio) were calculated according to Asare et al. [25]. Moreover, TG/HDL-C and oxLDL-C/HDL-C were quantified.

The ratio of LH to FSH was also calculated, as it is commonly increased in PCOS patients and linked to disturbances in ovulation [26].

### 2.5. Statistical Analysis

We performed statistical analysis using the Statistica Software Package, version 13.3 (Polish version; StatSoft, Kraków, Poland). Normality of variables was defined using the Shapiro–Wilk test, and homogeneity of variances was tested with Levene’s test. Depending on the results, we used Student’s t-test and the non-parametric U Mann–Whitney test to assess significant differences between subgroups divided according to age and the value of BMI, WHR, HOMA-IR or Quicki. The associations between oxidative stress parameters and lipids, glucose metabolism or hormone parameters were checked using Spearman’s rank-order correlation coefficient. The values were expressed as median, 1st quartile and 3rd quartile. In every analysis we considered the value of *p* < 0.05 as statistically significant.

## 3. Results

Measured parameters were examined in subgroups of patients divided according to age and the values of BMI, WHR, HOMA-IR and Quicki index. Characteristics of each subgroup are shown in Table 1 and Table 2. We found that women aged ≥25 years old had a significantly higher value of WHR and an unexpectedly lower concentration of oxLDL-C and lower value of oxLDL/HDL-C ratio when compared to women aged <25 years old. An increased BMI (≥25) value was associated with decreased concentration of HDL-C and an elevation in the concentration of TG, insulin (both fasting and after performing the OGTT), FRAP, the value of HOMA-IR and the ratios of CHO/HDL-C, LDL-C/HDL-C, TG/HDL-C and oxLDL-C/HDL-C when compared to women with a BMI <25.0. A higher value of waist-to-hip ratio (WHR ≥ 0.8) was linked to the increased concentrations of oxLDL-C, fasting glucose and insulin (both fasting and after performing the OGTT) and the value of TG/HDL-C ratio when compared to women with a WHR <0.8 (Table 1). More significant differences were found in the PCOS group divided according to Quicki value than the HOMA-IR index. Significant changes in the subgroup of women with a Quicki index <0.34 were detected in cases of WHR and HOMA-IR value, the concentration of HDL-C, insulin after the OGTT and values of CHO/HDL-C, LDL/HDL and oxLDL-C/HDL-C ratio. An elevation in the value of HOMA-IR (HOMA-IR ≥ 2.0) was associated with an increment in BMI, the concentration of fasting glucose and both concentrations of insulin (fasting and after performing the OGTT) when compared to the women with the value of HOMA-IR <2.0. (Table 2). 

Moreover, we revealed significant higher concentration of free T in the subgroup of women with WHR ≥0.8 when compared to women with WHR <0.8 (Table 3). Meanwhile, the concentration of LH and free T as well as the value of LH/FSH ratio were increased in women with Quicki <0.34 when compared to the women with a Quicki value ≥0.34 (Table 4). 

### Correlation Coefficients

We analyzed the dependencies between the FRAP value and oxLDL-C level and selected parameters in all subgroups. Results are shown in Table 5. The main factor associated with changes in the concentration of oxLDL-C and FRAP value was WHR. A positive correlation between the concentration of oxLDL and the value of WHR was observed in the whole studied group as well as in the subgroup of women aged <25 years; with WHR <0.8 and Quicki value <0.34, there was a strong positive correlation between the value of WHR and FRAP in the group of women with a BMI ≥25.0 and WHR ≥0.8. Furthermore, a higher BMI was related to an increment in the concentration of oxLDL-C and the value of FRAP in the whole studied group and in the subgroup of women with a WHR <0.8 and Quicki >0.34. A quite unexpected relationship was detected between age and the concentration of oxLDL-C. This significant correlation was found in the subgroup of young women (aged < 25 years), with abdominal obesity (WHR value ≥ 0.8) and insulin resistance assessed according to the value of the Quicki index (<0.34). The highest inverse coefficient correlation was revealed in the subgroup of women with the value of Quicki <0.34. In women aged <25 years old, we found an inverse correlation between fasting glucose concentration and FRAP value, whereas a positive correlation was observed in the subgroup of women with HOMA-IR <2.0 (between FRAP value and fasting glucose concentration) and with Quicki <0.34 (between FRAP value and the concentration of glucose after performing the OGTT).

The concentration of oxLDL and the value of FRAP correlated significantly with selected hormones. The concentration of AMH was negatively associated with the value of FRAP in the women ≥25 years old and with the concentration of oxLDL-C in women with abdominal obesity (WHR ≥ 0.8). In addition, an inverse relationship was revealed between the value of FRAP and the concentration of LH in the women with a BMI ≥25.0 or FSH in the women with a WHR <0.8. In the subgroup of women with a WHR <0.8, a positive correlation between the value of FRAP and LH/FSH ratio was also found. The concentration of AD was only significantly correlated with the value of FRAP in the subgroup of women with a BMI ≥25.0 (Table 5).

## 4. Discussion

Oxidative stress may not only be an important developmental cause of PCOS, but might also enhance symptoms in the course of this disorder [27]. On the other hand, many PCOS symptoms may intensify oxidative stress [18]. As we reported in our own previous study [14], disturbances in sex hormone concentration in women with PCOS could be associated with disorders in the lipid profile; therefore, in the present study, we chose the determination of oxLDL-C as an oxidative stress parameter, since it reflects the degrees of lipid peroxidation while helping to assess the disturbances in lipid profile parameters and the risk of developing cardiovascular diseases [28]. Furthermore, oxLDL-C induces inflammation within the arterial wall, which is involved in atherosclerotic plaque development [29]. Both low-grade chronic inflammation [30] and cardiovascular diseases [31] are common features of PCOS. An earlier conducted study confirmed that lipid peroxidation (expressed as serum malondialdehyde (MDA) concentration) was higher in women with than without PCOS [18,32], and the concentration was increased independent of age, weight or presence of insulin resistance [33]. Unexpectedly, our study observed that younger women with PCOS (<25 years old) had a higher concentration of oxLDL-C than women >25 years old. As a confirmation of this finding, we also found a significant negative correlation between age and the concentration of oxLDL in the subgroup of women with a BMI <25 (r = −0.33), with a WHR ≥0.8 (r = −0.75) and a Quicki <0.34 (r = −0.81). Moreover, we also revealed that the value of the oxLDL-C-C/HDL-C ratio was higher in younger women, but this index did not correlate with other investigated parameters. On the other hand, a higher concentration of oxLDL in the younger women with PCOS could be associated with a higher value of WHR [16]. Unfortunately, when we divided PCOS women according to age, we observed that the prevalence of abdominal obesity was more frequent in women <25.0 years old than in women ≥25.0, which may be the reason for the higher concentration of oxLDL in such patients.

We also detected a significantly higher concentration of oxLDL-C in the group with abdominal obesity. The value of the oxLDL-C/HDL-C ratio was higher in women who were overweight and had a Quicki index <0.34 when compared to women with a normal weight and Quicki ≥0.34, which clearly indicated that overweight/obesity had affected the concentration of oxLDL. In the study conducted by Demirel et al. [34], in the group of young women with PCOS, the authors showed the concentration of oxLDL-C in obese and nonobese adolescents with PCOS and in healthy controls to be similar. One explanation is that the mean age of patients and the control group was approximately 15 years old, and thus the duration time of PCOS was probably short, whereas in our study women with PCOS were almost two times older (27.0 ± 5.7 years old). Moreover, the authors did not observe any significant correlation between the concentration of oxLDL and BMI, hyperandrogenemia, lipids or insulin resistance in the studied group [34]. On the other hand, the study conducted by Oncul et al. [35] revealed an almost 2-fold higher concentration of oxLDL in the serum of women with PCOS. In addition, BMI value did not affect oxLDL concentration. Furthermore, other results indicated that body mass could significantly modulate the concentration of oxLDL-C in women not suffering from PCOS and revealed that weight loss of about 14% reduced the concentration of oxLDL by about 40% in the group of obese premenopausal women without PCOS [36]. It is possible that other important factors significantly altered oxLDL-C concentration in the course of PCOS.

To evaluate the pro/antioxidant balance, we also measured antioxidant capacity in women with PCOS. When we searched the data in PubMed, we mainly found inconsistent results of antioxidant status. Most studies show decreased total antioxidant status [37,38]. However, in a study conducted by Zhang et al. [39], the antioxidant capability of PCOS women was increased. In the present study, we focused on the effect of age, body mass and insulin resistance and sensitivity on the value of FRAP in different subgroups of women with PCOS. We used FRAP value, because not only does it reflect a single concentration/activity of an antioxidant, but it is also proportional to the molar concentration of the antioxidant capacity and reflects the total antioxidant capacity of plasma [40]. Murri et al. [18] published a systematic review and meta-analysis to evaluate the magnitude of oxidative stress in women with PCOS. This paper included a summary of six studies containing data about total antioxidant capacity; none revealed any statistically significant differences between women with and without PCOS. Generally, the antioxidant potential in women with PCOS is inconclusive. An almost 2-fold lower concentration of TAC and approximately 3-fold higher concentration of MDA was noticed in the group of PCOS patients compared with women without PCOS in the study conducted by Mohamadi et al. [41], whereas in the study conducted by Cakir et al. [42] no significant change in the concentration of total antioxidant status between women with and without PCOS was reached. Neither did they observe any significant correlation between TAS and serum androgens; therefore, they claimed that the determination of oxidative stress parameters is not useful in women with PCOS who are lean and in early age [43]. In the study by Carik et al. [42], the mean age and BMI was 23.6 ± 6.3 and 24.2 ± 4.0, respectively, whereas the women in the study conducted by Mohamadi et al. [41] were older and had higher body mass, with a mean age and BMI of 29.2 ± 3.0 and 28.1 ± 2.7, respectively. In our study, the mean value of age and BMI was 27.0 ± 5.7 and 24.0 ± 4.9, respectively, which indicated that the studied women were younger and leaner than in Mohamadi et al.’s study, and these differences may have affected the level of pro/antioxidant balance parameters. In turn, a higher level of total antioxidant status was found, and simultaneously an increased total antioxidant status in the serum of women with PCOS was also reported [43]. The investigated women were normoinsulinemic, young (mean age: 24.4 ± 4.1 years old) and lean (BMI: 21.2 ± 1.8), which could suggest that even if oxidative stress occurs, antioxidant capacity is sufficient for preventing oxidative damage. In the current study, we also detected that the concentration of oxLDL-C and the value of FRAP were positively correlated in the whole studied group and even stronger in the subgroup of women with an HOMA-IR <2.0, which could also confirm that insulin sensitive women with PCOS have sufficient antioxidant capacity to protect against oxidative stress. On the other hand, our study also enabled us to detect a positive relationship between the values of WHR and FRAP in women with abdominal obesity (WHR ≥ 0.8) or overweight/obese women (BMI ≥ 25.0), which could indicate that young age is especially responsible for sufficient antioxidant capacity. In addition, we found that alteration in glucose concentration also affected the value of FRAP. In the women aged <25 years old, we found an inverse correlation between glucose (fasting) concentration and FRAP value, whereas a positive correlation was observed in the subgroup of women with an HOMA-IR <2.0 (between FRAP value and fasting glucose concentration), and there was even a strong value of the correlation coefficient in the subgroup of women with a Quicki index <0.34 (between FRAP value and the concentration of glucose after performing the OGTT). The significant association between the level of oxLDL-C and FRAP with the value of BMI and WHR, as well as with glucose concentration, suggests similar findings to those obtained by Kanafchian et al. [44], in which the total antioxidant capacity in women with PCOS is not only linked to this disease, but could also be modulated by body weight and insulin resistance [44]. When we evaluated the relationship between the concentration of oxLDL-C and the value of FRAP with hormonal status, we observed that the concentration of FRAP was more significantly correlated with selected hormones than oxLDL-C. In the case of oxLDL-C concentration, we only found a strong inverse correlation with AMH concentration (r = −0.71; 0.007), whereas FRAP value was not only negatively associated with AMH concentration, but also with LH, FSH and AD levels. The concentration of AMH, which significantly correlated with oxLDL concentration and FRAP value, was proposed as a useful parameter for early diagnosis of PCOS [45]. The relationship between the concentration of AMH and insulin resistance in the course of PCOS is well documented [46]. However, in the case of AMH concentration and oxidative stress, we could not find any published correlations between AMH and pro/antioxidant balance. Oxidative stress and inflammation have also been suggested to intensify hyperandrogenemia [8], but in our study we only observed a significant correlation between the FRAP value and AD level, and not find any relationship with the concentration of total and free T or with DHEA-S.

Some limitations should be considered when interpreting these study results. Due to the small number of PCOS patients, the obtained results should be treated as preliminary. Moreover, the concentration of testosterone was assayed using an ELISA method instead of liquid chromatography–mass spectrometry, which should be also treated as a limitation of the study.

## 5. Conclusions

1. The main investigated factors that affected oxLDL concentration were age and abdominal obesity.
2. Both insulin resistance and disorders in pro/antioxidant balance intensified PCOS symptoms.
3. It seems that the Quicki index was better in the diversification of women with PCOS and insulin sensitive/resistance, and revealed more significant changes in pro/antioxidants than HOMA-IR.
4. The concentration of AMH was significantly associated both with oxLDL and FRAP concentrations.

## Figures and Tables

**Table 1:** Examined parameters in PCOS women divided according to age, BMI and WHR value.

SubgroupVariables	Age (Years)		BMI (kg/m^2^)		WHR	
	<25.0 Years	≥25.0 Years	<25.0	≥25.0	<0.8	≥0.8
	*n* = 18	*n* = 20	*n* = 30	*n* = 8	*n* = 23	*n* = 15
Age (years)	22.5 (20.0–25.0)	31.0 (28.0–34.0) *	26.0 (23.0–32.0)	25.5 (23.0–28.0)	26.0 (22.0–31.0)	28.0 (25.0–32.0)
BMI (kg/m^2^)	25.5 (21.5–27.3)	22.8 (21.2–24.2)	22.1 (21.0–23.5)	31.0 (30.1–31.9) ^#^	21.5 (19.8–23.5)	24.2 (23.4–31.2) ^●^
WHR	0.8 (0.8–0.9)	0.8 (0.7–0.8) *	0.8 (0.7–0.8)	0.9 (0.8–0.9) ^#^	0.8 (0.7–0.8)	0.9 (0.8–0.9) ^●^
CHO (mg/dL)	166.5 (140.0–189.0)	159.0 (146.0–178.0)	162.5 (143.0–172.0)	173.0 (151.0–197.0)	164.0 (146.0–181.0)	159.0 (133.0–178.0)
HDL-C (mg/dL)	62.6 (53.9–69.8)	58.3 (53.0–68.6)	63.8 (54.0–71.5)	49.3 (39.9–56.9) ^#^	65.8 (56.9–72.7)	51.7 (44.0–58.3)
LDL-C (mg/dL)	84.4 (72.8–106.6)	78.3 (68.6–87.7)	79.6 (68.6–93.3)	94.3 (78.3–116.2)	83.6 (68.6–93.3)	78.3 (68.1–106.6)
TG (mg/dL)	74.5 (58.0–100.0)	68.4 (63.4–117.0)	67.8 (61.3–80.0)	116.5 (82.8–198.0) ^#^	64.7 (58.0–80.0)	82.8 (64.5–188.0)
CHO/HDL-C	2.5 (2.4–3.2)	2.4 (2.2–3.1)	2.4 (2.2–2.8)	3.6 (2.4–4.6) ^#^	2.4 (2.2–2.6)	3.3 (2.3–3.7)
LDL/HDL	1.3 (1.1–1.8)	1.2 (1.0–1.6)	1.2 (1.0–1.6)	2.1 (1.2–2.5) ^#^	1.2 (1.0–1.4)	1.7 (1.1–2.3)
TG/HDL-C	1.2 (0.9–1.7)	1.1 (0.9–2.4)	1.0 (0.9–1.6)	2.3 (1.2–5.0) ^#^	1.0 (0.8–1.4)	1.6 (1.0–3.5) ^●^
oxLDL-C (U/dL)	10.2 (5.7–13.4)	5.5 (4.6–5.8) *	5.7 (4.6–9.6)	8.7 (5.6–14.0)	5.1 (4.3–11.4)	7.0 (5.7–9.6) ^●^
oxLDL-C/HDL-C (U/mg)	0.2 (0.1–0.2)	0.1 (0.1–0.1) *	0.1 (0.1–0.2)	0.2 (0.1–0.2) ^#^	0.1 (0.1–0.2)	0.2 (0.1–0.2)
Glucose 0′ (mg/dl)	83.7 (80.2–87.0)	84.4 (80.9–85.9)	84.6 (80.9–86.6)	81.9 (79.1–87.7)	84.4 (81.1–86.6)	84.9 (80.2–87.0)
Glucose 120′ (mg/dl)	109.0 (104.0–126.0)	108.0 (92.2–128.0)	108.0 (97.9–128.0)	115.5 (106.0–135.0)	104.0 (92.2–111.0)	126.0 (109.0–141.0) ^●^
Insulin 0′ (µIU/mL)	8.4 (4.8–11.7)	7.1 (4.5–10.5)	7.0 (4.3–9.6)	12.3 (10.3–13.9) ^#^	7.1 (4.3–9.6)	11.4 (4.5–13.3) ^●^
Insulin 120′ (µIU/mL)	50.3 (40.5–84.2)	46.8 (39.8–76.8)	41.4 (33.8–59.4)	91.8 (66.1–109.0) ^#^	40.7 (30.3–53.2)	89.6 (58.7–106.0) ^●^
HOMA-IR index	1.8 (0.9–2.3)	1.5 (0.9–2.1)	1.5 (0.9–2.0)	2.5 (2.1–2.8) ^#^	1.5 (0.9–2.0)	2.3 (0.9–2.8)
Quicki index	0.35 (0.34–0.39)	0.35 (0.34–0.39)	0.36 (0.34–0.39)	0.34 (0.33–0.34) ^#^	0.36 (0.34–0.39)	0.34 (0.33–0.39) ^●^
FRAP (mmol/L)	0.8 (0.7–0.9)	0.8 (0.7–0.9)	0.8 (0.7–0.9)	0.9 (0.8–1.0)	0.8 (0.7–0.9)	0.8 (0.7–0.9)

* *p* < 0.05 when compared to the subgroup under 25 years old; ^#^ *p* < 0.05 when compared to the subgroup with BMI <25; ^●^ *p* < 0.05 when compared to the subgroup with WHR <0.8. Legend: PCOS—polycystic ovary syndrome; BMI—body mass index; WHR—waist-to-hip ratio; CHO—cholesterol; HDL-C—high-density lipoprotein; LDL-C—low-density lipoprotein; TG—triglycerides; oxLDL—oxidized LDL-C; HOMA-IR—homeostatic model assessment for insulin resistance; Quicki—quantitative insulin sensitivity check index; FRAP—ferric reducing antioxidant power.

**Table 2:** Examined parameters in PCOS women divided according to HOMA-IR and Quicki value.

Variables	HOMA-IR Index		Quicki Index	
	<2.0	≥2.0	<0.34	≥0.34
	*n* = 23	*n* = 15	*n* = 11	*n* = 27
Age (years)	26.0 (23.0–33.0)	26.0 (22.0–29.0)	25.0 (22.0–30.0)	26.0 (23.0–32.5)
BMI (kg/m^2^)	22.0 (20.1–24.0)	23.7 (23.0–31.0) *	30.1 (23.5–31.9)	22.1 (21.1–24.0) ^#^
WHR	0.8 (0.7–0.8)	0.8 (0.8–0.9)	0.8 (0.8–0.9)	0.8 (0.7–0.8) ^#^
CHO (mg/dL)	164.0 (143.0–185.0)	161.0 (145.5–169.5)	163.0 (151.0–183.0)	162.5 (141.5–179.5)
HDL-C (mg/dL)	61.8 (53.7–72.7)	57.6 (46.3–66.8)	53.9 (39.9–68.1)	62.7 (54.2–71.7) ^#^
LDL-C (mg/dL)	83.7 (68.1–93.8)	78.3 (73.1–98.4)	85.1 (75.0–106.6)	79.6 (66.8–92.8)
TG (mg/dL)	68.1 (60.2–87.6)	78.5 (62.7–121.5)	82.8 (64.1–190.0)	67.8 (59.1–95.3)
CHO/HDL-C	2.4 (2.2–2.9)	2.5 (2.3–3.6)	3.2 (2.4–4.6)	2.4 (2.2–2.9) ^#^
LDL/HDL	1.3 (1.0–1.6)	1.3 (1.1–2.3)	1.8 (1.2–2.5)	1.2 (1.0–1.6) ^#^
TG/HDL-C	1.0 (0.9–1.6)	1.4 (1.0–2.3)	1.6 (1.1–5.0)	1.0 (0.9–1.6)
oxLDL-C (U/dL)	5.7 (4.7–9.6)	6.3 (4.8–12.3)	7.6 (5.6–13.4)	5.7 (4.6–9.6)
oxLDL-C/HDL-C (U/mg)	0.1 (0.1–0.2)	0.2 (0.1–0.2)	0.2 (0.1–0.2)	0.1 (0.1–0.1) ^#^
Glucose 0′ (mg/dl)	81.8 (79.5–84.9)	86.5 (81.9–88.4) *	86.5 (81.1–89.1)	82.3 (80.2–86.2)
Glucose 120′ (mg/dl)	104.0 (92.2–126.0)	110.5 (107.5–134.0)	125.0 (109.0–135.0)	107.0 (97.1–123.5)
Insulin 0′ (µIU/mL)	4.8 (4.2–7.1)	11.5 (10.2–13.1) *	12.3 (11.4–13.5)	6.1 (4.3–8.4) ^#^
Insulin 120′ (µIU/mL)	41.1 (30.3–53.1)	80.6 (53.5–103.0) *	93.9 (76.8–109.0)	41.0 (32.2–53.4)
HOMA-IR index	0,9 (0.8–1.5)	2.3 (2.1–2.7)	2.6 (2.3–2.9)	1.3 (0.8–1.8) ^#^
Quicki index	0.39 (0.36–0.4)	0.34 (0.33–0.34) *	0.33 (0.33–0.34)	0.37 (0.35–0.4) ^#^
FRAP (mmol/L)	0.8 (0.7–0.9)	0.8 (0.8–0.9)	0.8 (0.7–0.9)	0.8 (0.7–0.9)

* *p* < 0.05 when compared to the subgroup with HOMA-IR <2.00; ^#^ *p* < 0.05 when compared to the subgroup with Quicki <0.34. Legend: BMI—body mass index; WHR—waist-to-hip ratio; CHO—cholesterol; HDL-C—high-density lipoprotein; LDL-C—low-density lipoprotein; TG—triglycerides; oxLDL—oxidized LDL-C; HOMA-IR—homeostatic model assessment for insulin resistance; Quicki—quantitative insulin sensitivity check index; FRAP—ferric reducing antioxidant power.

**Table 3:** Hormone status in the studied groups divided according to age, BMI and WHR value.

Variables	Age (Years)		BMI (kg/m^2^)		WHR	
	<25.0 Years	≥25.0 Years	<25.0	≥25.0	<0.8	≥0.8
	*n* = 18	*n* = 20	*n* = 30	*n* = 8	*n* = 23	*n* = 15
LH (lU/L)	6.8 (6.0–9.6)	6.6 (5.7–8.4)	6.6 (5.4–8.4)	8.9 (6.7–9.6)	6.6 (5.4–8.0)	8.9 (6.1–12.0)
FSH (lU/L)	6.5 (5.4–8.4)	6.4 (5.4–7.0)	6.3 (5.4–7.7)	6.5 (5.4–8.0)	6.4 (5.4–8.4)	6.5 (5.4–7.9)
LH/FSH	1.1 (0.9–1.3)	1.1 (0.9–1.4)	1.0(0.8–1.3)	1.2 (1.1–1.5)	1.0 (0.8–1.3)	1.2 (1.0–1.5)
total T (ng/mL)	0.2 (0.2–0.3)	0.2 (0.2–0.3)	0.2 (0.2–0.3)	0.2 (0.2–0.3)	0.2 (0.2–0.3)	0.2 (0.2–0.4)
free T (pg/mL)	1.9 (1.3–2.4)	1.5 (1.3–2.4)	1.5 (1.3–2.3)	2.5 (1.7–2.9)	1.5 (1.2–1.8)	2.4 (2.2–3.3) ^●^
AD (ng/mL)	2.7 (2.0–3.0)	2.2 (1.8–3.1)	2.2 (1.8–3.1)	2.9 (2.0–3.1)	2.2 (1.8–3.0)	2.9 (1.9–3.2)
AMH (ng/mL)	6.1 (5.5–7.1)	5.2 (4.7–6.1)	5.4 (4.8–6.3)	6.1 (5.8–7.0)	5.3 (4.0–7.1)	6.1 (5.3–7.0)
DHEA-S (µg/mL)	276.0 (237.0–319.0)	331.0 (223.0–420.0)	291.0 (240.0–414.0)	237.0 (184.0–386.0)	292.0 (245.0–417.0)	271.0 (203.0–390.0)
TSH (uIU/mL)	1.9 (1.7–2.2)	1.8 (1.3–2.3)	1.9 (1.3–2.3)	2.0 (1.8–2.1)	1.9 (1.5–2.3)	2.0 (1.2–2.4)
17-OHP (ng/mL)	0.6 (0.5–0.8)	0.5 (0.4–0.7)	0.6 (0.4–0.8)	0.6 (0.4–0.7)	0.6 (0.4–0.8)	0.6 (0.4–0.8)
Prolactin (ng/mL)	11.8 (9.0–13.7)	10.5 (8.6–16.1)	10.5 (8.6–13.6)	11.8 (9.7–18.1)	10.4 (7.5–15.9)	11.8 (9.7–15.9)

^●^*p* < 0.05 when compared to the subgroup with WHR <0.8. Legend: BMI—body mass index; WHR—waist-to-hip ratio; LH—luteinizing hormone; FSH—follicle-stimulating hormone; total T—total testosterone; free T—free testosterone; AD—androstenedione; AMH—anti-Müllerian hormone; DHEA-S—dehydroepiandrosterone sulfate; TSH—thyrotropin; 17-OHP—17-hydroxyprogesterone.

**Table 4:** Hormone status in the studied groups divided according to HOMA-IR and Quicki value.

Variables	HOMA-IR Index		Quicki Index	
	<2.0	≥2.0	<0.34	≥0.34
	*n* = 23	*n* = 15	*n* = 11	*n* = 27
LH (lU/L)	6.6 (5.5–8.0)	7.9 (5.9–12.2)	9.6 (6.9–15.3)	6.4 (5.4–6.9) ^#^
FSH (lU/L)	6.5 (5.4–8.4)	6.1 (4.6–7.5)	6.6 (5.5–8.0)	6.2 (5.0–7.4)
LH/FSH	1.0 (0.8–1.2)	1.0 (0.8–1.2)	1.2 (1.1–2.4)	1.0 (0.8–1.2) ^#^
total T (ng/mL)	0.2 (0.2–0.3)	0.3 (0.2–0.4)	0.2 (0.2–0.4)	0.2 (0.2–0.3)
free T (pg/mL)	1.5 (1.3–2.2)	2.4 (1.5–2.8)	2.7 (1.5–3.3)	1.5 (1.3–2.2) ^#^
AD (ng/mL)	2.1 (1.8–2.7)	3.0 (2.3–3.5)	2.9 (2.6–3.2)	2.1 (1.8–3.0)
AMH (ng/mL)	5.3 (4.0–7.0)	5.9 (5.4–7.0)	6.1 (5.6–7.1)	5.3 (4.7–6.6)
DHEA-S (µg/mL)	290.0 (223.0–417.0)	279.5 (238.5–402.0)	244.0 (203.0–386.0)	296.0 (236.5–418.5)
TSH (uIU/mL)	1.9 (1.3–2.3)	1.9 (1.3–2.4)	1.9 (1.2–2.4)	1.9 (1.3–2.3)
17-OHP (ng/mL)	0.5 (0.4–0.7)	0.7 (0.4–0.9)	0.6 (0.4–0.8)	0.6 (0.4–0.8)
Prolactin (ng/mL)	10.5 (7.5–13.7)	10.5 (9.7–15.9)	10.3 (9.4–15.9)	10.8 (8.3–16.1)

^#^*p* < 0.05 when compared to the subgroup with Quicki <0.34. Legend: HOMA-IR—homeostatic model assessment for insulin resistance; Quicki—quantitative insulin sensitivity check index; LH—luteinizing hormone; FSH—follicle-stimulating hormone; total T—total testosterone; free T—free testosterone; AD—androstenedione; AMH—anti-Müllerian hormone; DHEA-S—dehydroepiandrosterone sulfate; TSH—thyrotropin; 17-OHP—17-hydroxyprogesterone.

**Table 5:** Correlation coefficient between the concentration of oxLDL or FRAP and studied parameters in subgroups of PCOS women.

Correlation Coefficient	oxLDL (U/dL)	FRAP (mmol/L)
**Whole Group, *n* = 38**		
BMI (kg/m^2^)	0.31; 0.044	0.32; 0.049
WHR	0.42; 0.009	NS
FRAP (mmol/L)	0.32; 0.049	NS
**Age < 25 years old, *n* = 18**		
WHR	0.48; 0.049	NS
glucose 0′ (mg/dL)	NS	−0.55; 0.18
**Age ≥ 25 years old, *n* = 20**		
AMH (ng/mL)	NS	−0.44; 0.044
**BMI < 25, *n* = 30**		
Age (years)	−0.33; 0.033	NS
**BMI ≥ 25, *n* = 8**		
WHR	NS	0.83; 0.011
LH (lU/L)	NS	−0,85; 0.037
AD [ng/mL]	NS	−0.67; 0.049
**WHR < 0.8, *n* = 23**		
WHR	0.43; 0.039	NS
BMI (kg/m^2^)	NS	0.54; 0.008
FSH (lU/L)	NS	−0,46; 0.028
LH/FSH	NS	0.46; 0.029
**WHR ≥ 0.8, *n* = 15**		
Age (years)	−0.75; 0.001	NS
AMH (ng/mL)	−0.71; 0.007	NS
WHR	NS	0.63; 0.011
**HOMA-IR < 2.0, *n* = 23**		
glucose 0′ (mg/dL)	0.93; 0.000	0.45; 0.027
FRAP (mmol/L)	0.51; 0.011	NS
**HOMA-IR ≥ 2.0, *n* = 15**		
	NS	NS
**QUICKI < 0.34, *n* = 11**		
glucose 120′ (mg/dL)	NS	0.69; 0.019
Age (years)	−0.81; 0.002	NS
**QUICKI ≥ 0.34, *n* = 27**		
WHR	0.45; 0.18	NS
glucose 120′ (mg/dL)	0.93; 0.000	NS
BMI (kg/m^2^)	NS	0.47; 0.012

Legend: HOMA-IR—homeostatic model assessment for insulin resistance; Quicki—quantitative insulin sensitivity check index; LH—luteinizing hormone; FSH—follicle-stimulating hormone; SHBG—sex hormone-binding globulin; total T—total testosterone; free T—free testosterone; AD—androstenedione; AMH—anti-Müllerian hormone; DHEA-S—dehydroepiandrosterone sulfate; TSH—thyrotropin; 17-OHP—17-hydroxyprogesterone.

## Data Availability

The data presented in this study are available on request from the corresponding author.
